# Role of IL-12 Levels in Diagnosing Tuberculosis Among People Living with HIV Receiving Antiretroviral Therapy

**DOI:** 10.3390/ijms27135854

**Published:** 2026-06-29

**Authors:** Ashwini Shete, Manisha Ghate, Sandip Patil, Pallavi Shidhaye, Bharati Mahajan, Hiroko Iwasaki-Hozumi, Takashi Matsuba, Toshio Hattori

**Affiliations:** 1 ICMR Indian Council of Medical Research—National Institute of Translational Virology and AIDS Research (ICMR-NITVAR, Formerly National AIDS Research Institute), Pune 411026, India; ashete@nariindia.org (A.S.); mghate@nariindia.org (M.G.); spatil@nariindia.org (S.P.); pshidhaye@nariindia.org (P.S.); bmahajan.nari@gov.in (B.M.); 2 Research Institute of Health and Welfare, Kibi International University, Takahashi 716-0018, Japan; hiro_ihz@kiui.ac.jp; 3 School of Pharmaceutical Science, Kyushu University of Medical Sciences, Nobeoka 882-8508, Japan; matsubat@phoenix.ac.jp; 4 The Shizuoka Graduate University of Public Health, Shizuoka City 420-0881, Japan

**Keywords:** IL-12, HIV, tuberculosis, coinfection, cytokines, biomarkers

## Abstract

Human immunodeficiency virus and tuberculosis (HIV/TB) coinfection remains a major global health challenge. Immune dysregulation in HIV complicates TB diagnosis. The type of immune response mounted in tuberculosis is a key determinant in deciding the outcome of the infection. Hence, estimating immune markers is critical for developing diagnostic, monitoring and treatment approaches. A study was conducted to evaluate the diagnostic potential of host-based biomarkers in individuals with HIV/TB coinfection in comparison to HIV monoinfection receiving antiretroviral therapy. Host-based biomarkers were quantified using commercially available kits. Receiver operated curve (ROC) analysis was conducted to determine diagnostic performance. Routine investigations showed significantly raised ratios of neutrophils, monocytes, and platelets-to-lymphocytes and alkaline phosphatase levels in HIV/TB coinfection (AUC values > 0.76). Plasma galectin-9 and osteopontin levels had an AUC > 0.8. IFN-γ, TNF-α and IL-12 levels were also significantly raised (AUC values > 0.95) while levels of GM-CSF and IL-6 were significantly low in HIV TB coinfection. The ROC analysis revealed the highest diagnostic accuracy of IL-12, indicating its potential as an adjunct immunological biomarker in identifying TB among HIV-infected individuals. However, a large-scale prospective study is required to confirm the findings and to understand their role in predicting the development of tuberculosis disease in people living with HIV.

## 1. Introduction

According to the WHO Global Tuberculosis Report 2025, TB remains the leading killer of people living with HIV (PLHIV), accounting for about 150,000 HIV-associated TB deaths in 2024. While the overall TB incidence is starting to fall due to widespread antiretroviral therapy (ART), people with HIV are 12 times more likely to fall ill with TB [1]. PLHIVs have a three times increased risk of mortality and morbidity due to tuberculosis in comparison to HIV-uninfected individuals even when undergoing ART [1]. The prevalence of TB was reported up to 72% in PLHIVs in high-burden settings [2]. The incidence rate of tuberculosis was 4.39 cases (95%CI 3.86–5.00) per 100 person-years in PLHIVs undergoing first-line ART [3].

HIV infection significantly impairs cell-mediated immunity, increasing susceptibility to active TB and complicating diagnosis. The development of tuberculosis disease and its outcomes largely depends on the type of immune response mounted by the host. The outcome of tuberculosis (TB) depends largely on the delicate balance between proinflammatory and anti-inflammatory cytokines secreted by immune cells upon recognition of mycobacterial antigens driving the development of protective or pathological immune responses [4]. Hence, cytokine profiles are investigated in multiple studies to develop novel treatments and diagnostic approaches [5].

Since HIV infection also poses challenges in terms of the diagnosis of tuberculosis, the discovery of novel biomarkers is of utmost essential for early diagnosis and prompt treatment initiation for better patient management as well as for curbing the secondary transmission of the infection [6,7]. Although pathogen-based biomarkers are desirable, they pose challenges in smear-negative pulmonary tuberculosis (PTB) and extrapulmonary tuberculosis (EPTB), which often require invasive techniques for sample collection. Hence, host-based biomarkers are investigated for the early detection of tuberculosis as well as for monitoring response to treatment. However, levels of the host-based biomarkers may be altered in HIV infection and are likely to depend on the level of immunosuppression in these individuals. Hence, data from the HIV-uninfected population cannot be used for predicting the effectiveness of various host-based biomarkers in predicting tuberculosis disease or treatment response in PLHIVs. Biomarkers identified in animal models may not always translate effectively to human disease due to differences in host–pathogen interactions and immune responses. Therefore, the identification and validation of biomarkers directly in human HIV/TB-coinfected populations are critical for developing clinically relevant diagnostic tools that can facilitate early diagnosis and timely treatment initiation. Apart from the diagnostic and prognostic potential of the host-based biomarkers, they might also be beneficial in ruling out TB infection prior to initiating preventive therapy for tuberculosis in PLHIVs.

Levels of host-based biomarkers need to be established independently in PLHIVs as low CD4 counts impair typical immune responses and might affect the performance of the diagnostic tests [8]. Although ART results in immune restoration and increased CD4 counts, not all the immune parameters reach their normal values. The WHO’s intensified case-finding screen for PLHIVs was reported to have poorer sensitivity in identifying TB in ART-treated individuals [9], indicating a need for suitable tests focusing on screening these individuals for TB. Such tests are recommended to have a sensitivity greater than 90% and a specificity greater than 70% as per the WHO consensus-gathering meeting for a rapid, non-sputum-based diagnostic test for pulmonary TB [10]. Given the immunological interplay between HIV, ART and TB, identifying reliable biomarkers for early TB detection in HIV-infected patients is crucial. The present study investigated the diagnostic potential of host-derived biomarkers in individuals with HIV/TB coinfection compared with those with HIV monoinfection receiving antiretroviral therapy (ART). The objective was to identify biomarker signatures associated with active tuberculosis in people living with HIV and to assess their potential role as adjunctive tools for improving TB diagnosis in this vulnerable population.

## 2. Results

### 2.1. Demographic Characteristics

The median ages of participants from group I and II were 47 (IQR: 45–51) and 46 (IQR: 37–50) years, with no statistically significant difference between groups (*p* > 0.05) (Table 1). The gender distribution was 36:34 in group I and 16:5 in group II, indicating a larger proportion of male participants in group II. CD4 counts in group I (median: 611; IQR: 448–731) were significantly higher than those in group II (median: 363; IQR: 198–510) participants, as expected.

### 2.2. Comparison of Host-Based Biomarker Levels in PLHIVs with and Without Tuberculosis

Significant differences were observed in the levels of the biomarkers presented in Table 2 except the absolute lymphocyte count between PLHIVs with and without tuberculosis (*p* < 0.05). Levels of aspartate aminotransferase, alanine aminotransferase, serum creatinine, blood urea nitrogen, and total and indirect bilirubin did not show significant differences between these groups. The biomarkers were also compared in HIV/TB-coinfected individuals before and after completing antituberculosis treatment using the Wilcoxon signed-rank test. The levels of the biomarkers, fold change in their levels after treatment and *p*-values for the comparisons are shown in Table 2.

### 2.3. Diagnostic Performance of Various Biomarkers for Detecting Tuberculosis in PLHIVs

ROC analysis was performed to determine diagnostic performance of the biomarkers by comparing their levels in HIV/TB coinfection versus in HIV monoinfection. The findings of the ROC analysis of the biomarkers for distinguishing HIV/TB coinfection from HIV monoinfection are shown in Table 3.

### 2.4. Correlation of Different Biomarkers with Each Other

**Figure 1:**
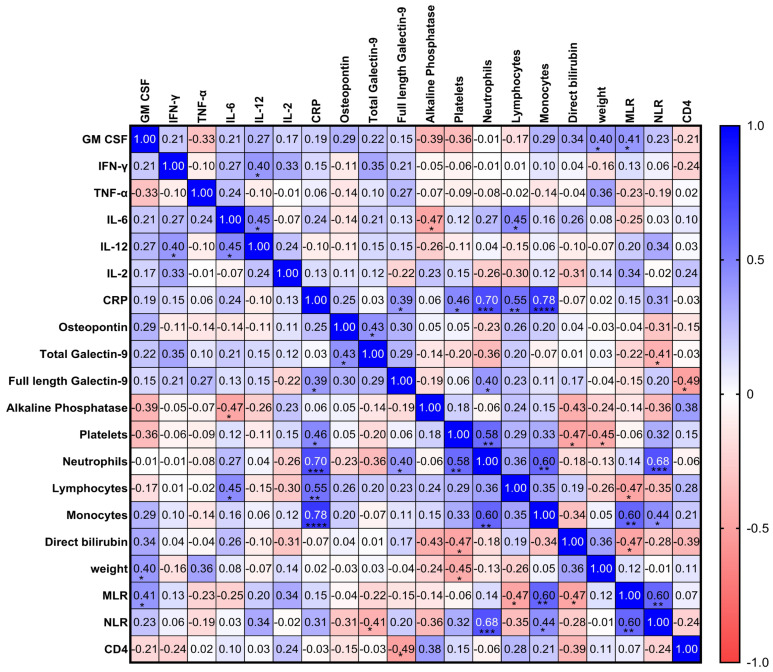
Double gradient heat map representing correlation analysis of immune parameters in HIV/TB coinfection: The heat map shows correlation analysis between various immune parameters in HIV/TB coinfection. The correlation coefficient (Spearman r) values are shown in each cell. The shades of blue represent positive correlation (r > 0), while the shades of red represent negative correlation (r < 0), as indicated in the scale shown in the figure. Significant *p* values are indicated by asterisks as follows: * (*p* < 0.05); ** (*p* < 0.01), *** (*p* < 0.001), **** (*p* < 0.0001).

Correlation analysis of various host-based biomarkers with each other was performed in HIV/TB-coinfected individuals, and the data are shown in Figure 1. A significant positive correlation was observed for GM-CSF with weight and MLR (r = 0.40 and 0.41, *p* = 0.035 and 0.046, respectively); IFN-γ with IL-12 (r = 0.40, *p* = 0.036); IL-6 with IL-12 and lymphocyte count (r = 0.45 for both, *p* = 0.02 and 0.03, respectively); hs-CRP with full-length galectin-9, platelets, neutrophils, lymphocytes and monocytes (r = 0.39, 0.46, 0.69, 0.55, 0.78; *p* = 0.04, 0.02, 0.0005, 0.008, <0.0001, respectively); total galectin-9 with osteopontin (r = 0.43, *p* = 0.03); full-length galectin-9 with neutrophils (r = 0.40, *p* = 0.04); platelets with neutrophils (r = 0.58, *p* = 0.004); neutrophils with monocytes (r = 0.6, *p* = 0.004); monocytes with MLR and NLR (r = 0.6 and 0.44, *p* = 0.004 and 0.03, respectively); and MLR with NLR (r = 0.6, *p* = 0.005).

A significant negative correlation was observed for the following: IL-6 with alkaline phosphatase (r = −0.47, *p* = 0.02); total galectin-9 with NLR (r = −0.41, *p* = 0.04); full-length galectin-9 with CD4 (r = −0.49, *p* = 0.01); platelets with direct bilirubin and weight (r = −0.47 and −0.44, *p* = 0.03 and 0.02, respectively); lymphocytes with MLR (r = −0.47, *p* = 0.02); and direct bilirubin with MLR (r = −0.47, *p* = 0.04).

### 2.5. Effect of Successful Antituberculosis Therapy on the Levels of the Biomarkers

Follow-up blood samples from the participants with HIV/TB coinfection were collected at the end of the antituberculosis therapy to test the biomarkers. The levels of these biomarkers and their comparison with the baseline levels are shown in Table 2. Accordingly, the biomarkers which decreased significantly after antituberculosis treatment were direct bilirubin (*p* = 0.005), platelets (*p* = 0.005), NLR (*p* = 0.0353), CRP (*p* = 0.0153), osteopontin (*p* = 0.0073), and full-length galectin-9 (*p* = 0.0007). The weight of the participants increased significantly after the antituberculosis treatment (*p* = 0.014). There were no significant differences in the levels of the other biomarkers.

## 3. Discussion

Although ART significantly reduces the risk of tuberculosis in PLHIVs, they are still reported to have higher incidence and mortality rates due to tuberculosis. Early detection and prompt treatment are required for better management. Hence, this study was conducted to determine the levels of various parameters in PLHIVs on ART to assess their potential as biomarkers for the possible diagnosis of tuberculosis in this population. The biomarkers included routine haematological and biochemical as well as immunological parameters.

Significant differences were observed in haematological parameters such as absolute neutrophil, monocyte and platelet counts, all of which were higher in HIV/TB coinfection. NLR, MLR and PLR, which have been reported to serve as the possible biomarkers of tuberculosis disease, also differed significantly in them with bigger differences than the individual counts. The AUC for these haematological ratios differed between 0.71 and 0.77. The monocyte-to-lymphocyte ratio (MLR) and platelet-to-lymphocyte ratio (PLR) have been proposed as biomarkers for the detection of incident symptomatic TB in previous reports [11,12]. Increased neutrophils are known to mediate inflammatory damage in tuberculosis [13] with a higher neutrophil-to-lymphocyte ratio (NLR) reported in HIV/TB coinfection [14]. The NLR’s receiver operating characteristic (ROC) has been shown to perform better than CRP in diagnosing TB previously [15]. CRP levels strongly correlated positively with neutrophil and monocyte count in our study. Active tuberculosis infection is known to induce a systemic inflammatory response [16], creating a positive correlation between these markers. Meanwhile, lymphocyte count is often decreased especially in severe forms of tuberculosis [17], making the NLR and MLR more pertinent markers than the individual cell counts.

Among biochemical parameters, alkaline phosphatase and direct bilirubin levels were significantly higher in HIV/TB coinfection. The AUC for alkaline phosphatase levels was 0.795. The usefulness of alkaline phosphatase in differentiating tuberculous versus non-tuberculous etiologies for extrapulmonary manifestations such as pleural effusion/lymphadenitis has been demonstrated previously [18,19]. CRP is recognized as a screening marker for TB in PLHIVs in WHO guidelines. We observed an AUC of 0.924 with a cut-off of 0.57 mg/L, yielding both a sensitivity and specificity above 90%, indicating its strong tuberculosis diagnostic potential in PLHIVs receiving ART. A threshold of >5 mg/L for CRP was shown to have a sensitivity of more than 80% with a low specificity, which was below 50% [20]. A study conducted in Uganda showed an 89% sensitivity and 72% specificity of the CRP test for diagnosis of culture-confirmed TB in PLHIVs commencing anti-retroviral therapy (ART) and having CD4 levels equal to or less than 340 cells/mL [21]. The use of published cut-offs for CRP and also MLR for detecting TB in individuals with and without HIV infection was reported to have a poor sensitivity and positive predictive value [22]. They reported a lower cut-off of >3.3 mg/L for CRP with a sensitivity of 80% and a specificity of 72%, which matched the optimum criteria recommended by WHO for the target product profile for tuberculosis diagnostic tests [22].

The cytokine levels investigated in the study showed significantly higher levels of IFN-γ, TNF-α, IL-12 and IL-2 in HIV/TB coinfection. The AUC values for these cytokines, except for IL-2, were above 0.95, indicating their potential to serve as biomarkers for diagnosing tuberculosis. Higher plasma levels of Th1 cytokines such as IFN-γ and TNF-α in tuberculosis have been reported previously [23]. The reduced IL-12 in HIV-infected individuals reflects impaired antigen-presenting cell function. The elevated IL-12 in TB patients aligns with the expected Th1 immune response to M. tuberculosis. It was observed that TB cases had 13.01-fold higher IL-12 mRNA expression than healthy controls [24]. IL-12 is a heterodimeric cytokine produced primarily by dendritic cells and macrophages. It plays a pivotal role in promoting Th1 differentiation and IFN-γ production, both of which are critical for the containment of *Mycobacterium tuberculosis*. HIV infection disrupts cytokine networks, including IL-12 production, potentially impairing anti-mycobacterial immunity.

Surprisingly, levels of GM-CSF and IL-6 were significantly lower in HIV/TB coinfection, although IL-12 and IL-6 levels correlated positively with each other, suggesting other mechanisms for the downregulation of IL-6 levels. IL-6 levels were negatively correlated with alkaline phosphatase levels. Alkaline phosphatase has been known to possess anti-inflammatory activity, reducing levels of proinflammatory cytokines such as IL-6 [25]. Costimulation of IL-6 axis was reported to result in the significant induction of alkaline phosphatase activity, which was reduced back to baseline levels after blocking IL-6 by sarilumab in one of the studies [26]. GM-CSF levels also correlated with TNF-α levels and platelet count. GM-CSF levels secreted by macrophages or PBMCs of active TB patients were reported to be significantly low and have demonstrated a reduced capacity to control the intracellular growth of M. tuberculosis in previous studies [27,28].

Levels of matricellular proteins such as osteopontin and galectin-9 were also significantly raised in HIV/TB coinfection. We previously investigated their role as diagnostic markers in HIV/TB coinfection and in PLHIVs not undergoing ART [29]. The AUC values (0.9 vs. 0.86) and Youden index (0.6 vs. 0.5) were diminished in ART-experienced PLHIVs in the current study for galectin-9 levels, while these values improved for osteopontin levels with higher AUC values (0.76 vs. 0.83) and a greater Youden index score (0.5 vs. 0.6) as compared to ART-naïve PLHIVs studied previously. ART is responsible for immune restoration, altering levels of immune parameters, and hence, it is critical to establish a differential cut-off in PLHIVs with and without ART. We observed interesting, contradictory correlations between full-length galectin-9 and neutrophils, whose correlation was positive, while that for total galectin-9 and NLR was negative. Neutrophils have been shown to release more galectin-9 in PLHIVs with low CD4 counts [30]. This might be responsible for the negative correlation observed in our study between full-length galectin-9 and CD4 counts. Neutrophil-derived galectin-9 acts as a critical immune regulator, in turn inhibiting MMP-2 and 9, which mediate its breakdown, possibly reducing total galectin-9 levels [31].

This study has several limitations, such as its small sample size, the exploratory cut-offs of the biomarkers derived only within the study population, selection bias while enrolling the participants, absence of definitive tuberculosis diagnosis for EPTB cases and multiple statistical comparisons increasing the risk of type I error. In addition, factors such as CD4 cell count, HIV disease severity, antiretroviral therapy status, time lapse between baseline sample collection and antituberculosis treatment initiation, and other clinical variables may influence biomarker levels. Finally, the study was conducted in a specific clinical and geographic setting, which may limit the generalizability of the findings to other populations. Future larger, prospective, multicenter studies with predefined thresholds and external validation are needed to confirm these results. Also, the performance of the host-based biomarkers may differ in adult and pediatric populations [32]. Therefore, findings from studies conducted using adults cannot be directly extrapolated to children.

## 4. Materials and Methods

### 4.1. Study Groups and Sample Collection

A study for cross-sectional diagnostic comparison with a longitudinal follow-up component was performed by enrolling participants from ART centres in Pune city after obtaining their informed consent. Two study groups were Group I: HIV-positive patients on ART without active TB (*n* = 70) and Group II: HIV/TB-coinfected patients detected after ART initiation (*n* = 21). The data analyzed from HIV/TB-coinfected individuals irrespective of their ART status for monitoring response to antituberculosis treatment was published previously [33,34]. A new analysis was performed for ART-experienced individuals and compared with ART-experienced HIV-infected controls without active tuberculosis. Inclusion criteria for group I included the following: patients who were ≥18 years old, HIV infected and taking antiretroviral treatment, virally suppressed, asymptomatic at the time of enrollment, and free of any active acute or chronic co-infections. Inclusion criteria for group II included the following: patients who were ≥18 years old, HIV infected and taking antiretroviral treatment, co-infected with TB, and either being initiated for anti-tubercular treatment or within 7 days of initiation. Exclusion criteria for both groups included PLHIVs younger than 18 years and those who were inable to communicate and/or in a physical or mental state that made them unable to provide informed consent. HIV diagnosis was confirmed by standard 3-test HIV testing algorithm as per the national AIDS control program guidelines. Adults with active PTB (*n* = 12) were microbiologically confirmed by sputum acid-fast bacillus test (AFB) and/or Xpert MTB/RIF assay. The diagnosis of EPTB (*n* = 9) was primarily presumptive, based on clinical signs and symptoms. Radiological investigations were performed whenever indicated. TB diagnosis was based on sputum smear microscopy, GeneXpert MTB/RIF assay and/or clinical findings. Blood samples for group I were collected once at the time of enrollment, while blood samples for group II were collected twice: before or within 7 days of antituberculosis treatment initiation and after completion of antituberculosis treatment. Among the 21 participants with HIV/TB coinfection, blood samples were collected before ATT initiation in 9 participants, within 3 days of ATT initiation in 7 participants, and after 4 days of ATT initiation in 5 participants. Plasma was separated, aliquoted to avoid frequent freezing–thawing and stored at −80 °C until analysis.

### 4.2. Routine Investigation

Routine tests for complete blood count, CD4 count and percentage, aspartate aminotransferase, alanine aminotransferase, alkaline phosphatase, total, direct, and indirect bilirubin, serum creatinine and blood urea nitrogen were performed at NABL-accredited laboratories.

### 4.3. Biomarker Measurement

Commercially available kits, namely Human Osteopontin DuoSet ELISA Kits (R&D Systems, Minneapolis, MN, USA), Human Galectin-9 DuoSet ELISA Kits (R&D Systems, Minneapolis, MN, USA), Human GAL9 ELISA Kit (ELISA Genie, Dublin, Ireland), High Sensitivity C-Reactive Protein Enzyme Immunoassay Test Kit (Bio Check, Scarborough, ME, USA), were used for estimating matricellular protein and CRP levels in plasma samples. Commercially available customized multiplex luminex based assays (Merck Millipore, Burlington, MA, USA) were performed to estimate levels of cytokine such as GM-CSF, interferon-gamma (IFN-γ), IL-2, TNF-α, IL-12 (p70) and IL-6 using Bio-Plex 200 system (Bio-Rad, Hercules, CA, USA). Kit details and performance characteristics are mentioned in Table 4. All the assays were performed as per the manufacturers’ instructions, and readings were taken using calibrated readers. The plasma cytokine levels below the minimum detectable level were assigned a value of 0 for statistical analysis.

### 4.4. Statistical Analysis

Data were analyzed using GraphPad Prism version 9.0. Quantitative variables were expressed as median ± interquartile ranges. Differences between groups were assessed using Mann Whitney test. Differences in the immune parameters before and after antituberculosis treatment were assessed using Wilkoxon signed-rank test. Receiver operating characteristic (ROC) curves were constructed to evaluate diagnostic accuracy. Youden index was calculated as sensitivity + specificity-1. A *p*-value < 0.05 was considered statistically significant. Spearman test was used to calculate r and *p* values in the correlation analysis.

## 5. Conclusions

Plasma IL-12 levels differed significantly among HIV and HIV/TB-coinfected patients and demonstrated a high diagnostic accuracy in identifying TB among HIV-infected individuals. Future longitudinal studies with larger cohorts are warranted to validate IL-12 as a clinically useful biomarker. Although these findings suggest IL-12 may serve as an adjunct biomarker for detecting TB in PLHIVs, the persistence of elevated IL-12 levels following anti-tuberculosis treatment suggests its limited utility for monitoring treatment response or predicting subsequent TB development in PLHIVs. Apart from reporting the diagnostic potential of IL-12 levels in HIV/TB coinfection, the study also highlighted the interesting associations among various host-based biomarkers, providing insights into the immune mechanisms involved in HIV/TB coinfection that may contribute to disease pathogenesis.

## Figures and Tables

**Table 1:** Demographic characteristics of the study participants.

Parameters, Median (IQR)	Group I: ART Experienced PLHIV Without Active TB (*n* = 70)	Group II: ART Experienced HIV/TB-Coinfected Cases (*n* = 21)	*p* Value
Age (Years)	47 (45–51)	46 (37–50)	NS
Gender—M:F	36:34	16:5	0.0492
CD4 count (cells/mL)	611 (448–731)	363 (198–510)	<0.0001

NS—Not significant.

**Table 2:** Levels of host-based biomarkers in PLHIVs with and without tuberculosis.

Biomarkers: Median (Interquartile Range)	Levels in HIV Monoinfection (Group I) (*n* = 70)	Levels in HIV/TB Coinfection Before Treatment (Group II) (*n* = 21)	Levels in HIV/TB Coinfection After Treatment (*n* = 21)	Fold Change in the Levels After Treatment	*p* Value (PLHIV with and Without TB)	*p* Value (HIV/TB-Coinfected Individuals Before and After Treatment)
Weight (kg)	61.45 (51.7–70.55)	47.3 (41.5–60)	55 (45.48–63)	1.082 (0.993–1.212)	0.0021	0.0140
Alkaline Phosphatase (IU/L)	94 (77–115)	190.4 (98.5–308.5)	93.2 (78.75–148)	0.744 (0.363–1.132)	<0.0001	NS
Direct Bilirubin (mg/dL)	0.1 (0.1–0.2)	0.2 (0.125–0.2925)	0.145 (0.1–0.3)	1 (0.624–1)	0.0019	0.005
Platelets (cells/µL)	274 (223.8–338.3)	359.5 (282.5–461.8)	232.5 (176–278.8)	0.728 (0.5–0.981)	0.0034	0.0054
Neutrophils (cells/µL)	3.38 (2.71–4.46)	4.3 (3.28–6.2)	3.175 (2.4–4.185)	0.776 (0.534–1.068)	0.0189	NS
Lymphocytes (cells/µL)	2.15 (1.77–2.8)	1.74 (1.39–2.69)	1.6 (1.323–2.023)	1.009 (0.883–1.156)	0.0952	NS
Monocytes (cells/µL)	0.33 (0.25–0.46)	0.47 (0.32–0.58)	0.35 (0.24–0.45)	0.758 (0.709–1.45)	0.0324	NS
Neutrophils to Lymphocytes Ratio (NLR)	1.54 (1.26–1.87)	2.74 (1.59–3.55)	1.79 (1.33–3.06)	0.817 (0.708–0.982)	0.0007	0.0353
Monocytes to Lymphocytes Ratio (MLR)	0.15 (0.12–0.2)	0.2 (0.17–0.35)	0.24 (0.11–0.35)	0.717 (0.631–1.444)	0.0003	NS
Platelets to Lymphocytes Ratio (PLR)	120.9 (93.9–160.5)	177.0 (138.4–253.6)	155.1 (113.3–174.5)	0.757 (0.526–0.929)	0.0001	0.0107
GM CSF (pg/mL)	0.65 (0–0.65)	0.16 (0.04–0.4)	0.11 (0–0.29)	0.008 (0–1.596)	0.0494	NS
IFN-γ (pg/mL)	0.13 (0–0.34)	11.07 (6.02–26.24)	7.74 (1.15–31.65)	0.688 (0.220–1.721)	<0.0001	NS
TNF-α (pg/mL)	2.42 (0.66–7.34)	71.45 (32.67–103.5)	66.08 (37.82–216)	1.046 (0.582–6.35)	<0.0001	NS
IL-6 (pg/mL)	1.24 (0.61–4.35)	0 (0–4.34)	0 (0–77.71)	0.518 (0–8.104)	<0.0001	NS
IL-12 (pg/mL)	0 (0–0)	4.55 (3.09–9.68)	4.59 (3.3–15.65)	1 (0.641–1.644)	<0.0001	NS
IL-2 (pg/mL)	0 (0–0)	6.61 (0–14.68)	7.9 (0–25.76)	0.284 (0–1.365)	<0.0001	NS
CRP (mg/L)	1.66 (0.91–3.99)	19.56 (8.56–73.74)	2.96 (0.84–8.39)	0.125 (0.063–0.379)	<0.0001	0.0153
Osteopontin (pg/mL)	47,040 (35,063–63,958)	85,455 (69,709–102,770)	47,255 (37,331–91,118)	0.618 (0.407–1.18)	<0.0001	0.0073
Total Galectin-9 (pg/mL)	3758 (3075–4787)	7122 (4640–9687)	4974 (3804–6658)	0.67 (0.547–1.135)	<0.0001	NS
Full-length Galectin-9 (pg/mL)	1517 (545.8–7895)	4418 (2125–20,000)	765.4 (218.1–2945)	0.22 (0.102–0.525)	0.0121	0.0007

NS—Not significant.

**Table 3:** Diagnostic performance of host-based biomarkers for detecting tuberculosis in PLHIVs.

Biomarkers	AUC	95% CI	Cut-Off (mg/L)	Sensitivity%	95% CI	Specificity%	95% CI	Likelihood Ratio	Youden Index
Alkaline phosphatase	0.795	0.672 to 0.918	>94.50	88.89	67.20% to 98.03%	52.38	40.27% to 64.22%	1.867	0.413
Platelets	0.713	0.566 to 0.859	>375.5	50	29.93% to 70.07%	91.43	82.53% to 96.01%	5.833	0.414
NLR	0.73	0.603 to 0.896	>1.970	73.68	51.21% to 88.19%	78.57	67.61% to 86.56%	3.439	0.5225
MLR	0.767	0.658 to 0.875	>0.166	88.89	67.20% to 98.03%	58.57	46.88% to 69.37%	2.146	0.4746
PLR	0.78	0.662 to 0.898	>133.6	89.47	68.61% to 98.13%	62.86	51.15% to 73.23%	2.409	0.5233
IFN-γ	0.965	0.916 to 1.000	>2.270	90.48	71.09% to 98.31%	100	94.80% to 100.0%		0.9048
TNF-α	0.982	0.958 to 1.000	>16.89	100	84.54% to 100.0%	94.29	86.21% to 97.76%	17.5	0.9429
IL-6	0.778	0.612 to 0.944	<0.015	76.19	54.91% to 89.37%	98.57	92.34% to 99.93%	53.33	0.7476
IL-12	0.998	0.993 to 1.000	>1.355	100	84.54% to 100.0%	98.57	92.34% to 99.93%	70	0.9857
IL-2	0.823	0.6945 to 0.9517	>2.590	66.67	45.37% to 82.81%	98.57	92.34% to 99.93%	46.67	0.6524
GM CSF	0.635	0.501 to 0.770	<0.635	85.71	65.36% to 95.02%	62.86	51.15% to 73.23%	2.308	0.4857
CRP	0.924	0.845 to 1.000	>5.719	90.48	71.09% to 98.31%	90.14	81.02% to 95.14%	9.177	0.8062
Osteopontin	0.837	0.743 to 0.931	>68,015	85.71	65.36% to 95.02%	78.87	68.03% to 86.76%	4.057	0.6458
Total Galectin-9	0.855	0.771 to 0.939	>4603	80.95	60.00% to 92.33%	71.83	60.46% to 80.96%	2.874	0.5278
Full-length Galectin-9	0.681	0.555 to 0.806	>2775	76.19	54.91% to 89.37%	66.13	53.72% to 76.66%	2.249	0.4232

**Table 4:** Assay specifications and analytical performance characteristics of immunologic biomarkers.

Analyte	Manufacturer	Kit Catalog Number	Sample Dilution	Limits of Detection
GM CSF	Merck Millipore, USA	HTH17 MAG14K-12	Neat	0.206 ng/mL
IFN-γ				2.4 pg/mL
TNF-α				1.7 pg/mL
IL-6				2.9 pg/mL
IL-12 (pg/mL)				2.2 pg/mL
IL-2 (pg/mL)				9 pg/mL
CRP	Bio Check, USA	BC-1119	1:100	0.1 mg/L
Osteopontin	R&D Systems, USA	DY1433	1:100	62.5 pg/mL
Total Galectin-9	R&D Systems, USA	DY20450	1:10	93.8 pg/mL
Full-length Galectin-9	ELISA Genie, Ireland	HUES02158	1:10	7.8 pg/mL

## Data Availability

All data are available upon request.

## Abbreviations

The following abbreviations are used in this manuscript:

HIV	Human immunodeficiency virus
PLHIVs	People living with HIV
ART	Antiretroviral therapy
TB	Tuberculosis
CRP	C-reactive protein
NLR	Neutrophils to lymphocytes ratio
MLR	Monocytes to lymphocytes ratio
PLR	Platelets to lymphocytes ratio
ROC	receiver operating characteristic
AUC	Area under curve
IQR	Interquartile ranges
IL	Interleukin
IFN	Interferon
TNF	Tissue necrosis factor
GM-CSF	Granulocyte-macrophage colony-stimulating factor

## References

[1] Global Programme on Tuberculosis and Lung Health (GTB) (2020). Global Tuberculosis Report 2020.

[2] Gao J., Zheng P., Fu H. (2013). Prevalence of TB/HIV co-infection in countries except China: A systematic review and meta-analysis. PLoS ONE.

[3] Gupte A.N., Kadam D., Sangle S., Rewari B.B., Salvi S., Chavan A., Nimkar S., Golub J., Gupte N., Gupta A. (2019). Incidence of tuberculosis in HIV-infected adults on first- and second-line antiretroviral therapy in India. BMC Infect. Dis..

[4] Domingo-Gonzalez R., Prince O., Cooper A., Khader S.A. (2016). Cytokines and Chemokines in Mycobacterium tuberculosis Infection. Microbiol. Spectr..

[5] Mihret A., Bekele Y., Bobosha K., Kidd M., Aseffa A., Howe R., Walzl G. (2013). Plasma cytokines and chemokines differentiate between active disease and non-active tuberculosis infection. J. Infect..

[6] Kroidl I., Ahmed M.I.M., Horn S., Polyak C., Esber A., Parikh A., Eller L.A., Kibuuka H., Semwogerere M., Mwesigwa B. (2022). Assessment of tuberculosis disease activity in people infected with Mycobacterium tuberculosis and living with HIV: A longitudinal cohort study. EClinicalMedicine.

[7] Sossen B., Kubjane M., Meintjes G. (2025). Tuberculosis and HIV coinfection: Progress and challenges towards reducing incidence and mortality. Int. J. Infect. Dis..

[8] Singer S.N., Ndumnego O.C., Kim R.S., Ndung’u T., Anastos K., French A., Churchyard G., Paramithiothis E., Kasprowicz V.O., Achkar J.M. (2022). Plasma host protein biomarkers correlating with increasing Mycobacterium tuberculosis infection activity prior to tuberculosis diagnosis in people living with HIV. EBioMedicine.

[9] Hamada Y., Lujan J., Schenkel K., Ford N., Getahun H. (2018). Sensitivity and specificity of WHO’s recommended four-symptom screening rule for tuberculosis in people living with HIV: A systematic review and meta-analysis. Lancet HIV.

[10] World Health Organization (2014). High-Priority Target Product Profiles for New Tuberculosis Diagnostics: Report of a Consensus Meeting.

[11] Chen G., Wu C., Luo Z., Teng Y., Mao S. (2016). Platelet-lymphocyte ratios: A potential marker for pulmonary tuberculosis diagnosis in COPD patients. Int. J. Chronic Obstr. Pulm. Dis..

[12] Wang J., Yin Y., Wang X., Pei H., Kuai S., Gu L., Xing H., Zhang Y., Huang Q., Guan B. (2015). Ratio of monocytes to lymphocytes in peripheral blood in patients diagnosed with active tuberculosis. Braz. J. Infect. Dis..

[13] Nwongbouwoh Muefong C., Owolabi O., Donkor S., Charalambous S., Bakuli A., Rachow A., Geldmacher C., Sutherland J.S. (2022). Neutrophils Contribute to Severity of Tuberculosis Pathology and Recovery From Lung Damage Pre- and Posttreatment. Clin. Infect. Dis..

[14] Sulastri N., Alisjahbana B., Livia R., Sahiratmadja E. (2021). Higher Neutrophil-lymphocyte Ratio in TB/HIV Co-infection Compared to Pulmonary Tuberculosis. Indones. Biomed. J..

[15] Yoon N.B., Son C., Um S.J. (2013). Role of the neutrophil-lymphocyte count ratio in the differential diagnosis between pulmonary tuberculosis and bacterial community-acquired pneumonia. Ann. Lab. Med..

[16] Zhai Y., Ren J., Ding Z., Xu F., Qu S., Bian K., Chen J., Yao M., Yao F., Liu B. (2024). The diagnostic value of hydroxyproline combined with tuberculosis infection T lymphocyte spot assay in pulmonary tuberculosis. J. Thorac. Dis..

[17] Li F., Chen D., Zeng Q., Du Y. (2023). Possible Mechanisms of Lymphopenia in Severe Tuberculosis. Microorganisms.

[18] Jadhav A.A., Bardapurkar J.S., Jain A. (2009). Alkaline phosphatase: Distinguishing between tuberculous and nontuberculous pleural effusion. Lung India.

[19] Kailas C.K., Srikantaiah H.C., Ashok A.C., Vinay B.M. (2016). Alkaline Phosphatase Levels in Tuberculous Cervical Lymphadenitis—Could it be a Predictor for Diagnosis and Monitoring Response to Anti-tuberculous Treatment. IJSS J. Surg..

[20] Reeve B.W., Ndlangalavu G., Mishra H., Palmer Z., Tshivhula H., Rockman L., Naidoo S., Mbu D.L., Naidoo C.C., Derendinger B. (2023). Point-of-care C-reactive protein and Xpert MTB/RIF Ultra for tuberculosis screening and diagnosis in unselected antiretroviral therapy initiators: A prospective diagnostic accuracy study. medRxiv.

[21] Yoon C., Semitala F.C., Atuhumuza E., Katende J., Mwebe S., Asege L., Armstrong D.T., Andama A.O., Dowdy D.W., Davis J.L. (2017). Point-of-care C-reactive protein-based tuberculosis screening for people living with HIV: A diagnostic accuracy study. Lancet Infect. Dis..

[22] Gersh J.K., Barnabas R.V., Matemo D., Kinuthia J., Feldman Z., Lacourse S.M., Mecha J., Warr A.J., Kamene M., Horne D.J. (2021). Pulmonary tuberculosis screening in anti-retroviral treated adults living with HIV in Kenya. BMC Infect. Dis..

[23] Bolajoko E.B., Arinola O.G., Odaibo G.N., Maiga M. (2020). Plasma levels of tumor necrosis factor-alpha, interferon-gamma, inducible nitric oxide synthase, and 3-nitrotyrosine in drug-resistant and drug-sensitive pulmonary tuberculosis patients, Ibadan, Nigeria. Int. J. Mycobacteriol..

[24] Abohashrh M., Ahmad I., Alam M.M., Beg M.M.A., Alshahrani M.Y., Irfan S., Verma A.K., Alshaghdali K., Saeed M. (2022). Assessment of IL-12, mRNA expression, vitamin-D level, and their correlation among the Mycobacterium tuberculosis cases. Saudi J. Biol. Sci..

[25] Singh S.B., Lin H.C. (2021). Role of Intestinal Alkaline Phosphatase in Innate Immunity. Biomolecules.

[26] Klinder A., Waletzko-Hellwig J., Sellin M.L., Seyfarth-Sehlke A., Wolfien M., Prehn F., Bader R., Jonitz-Heincke A. (2022). Effects of the Interleukin-6 Receptor Blocker Sarilumab on Metabolic Activity and Differentiation Capacity of Primary Human Osteoblasts. Pharmaceutics.

[27] Balcells M.E., Ruiz-Tagle C., Tiznado C., Garcia P., Naves R. (2018). Diagnostic performance of GM-CSF and IL-2 in response to long-term specific-antigen cell stimulation in patients with active and latent tuberculosis infection. Tuberculosis.

[28] Mishra A., Singh V.K., Jagannath C., Subbian S., Restrepo B.I., Gauduin M.C., Khan A. (2022). Human Macrophages Exhibit GM-CSF Dependent Restriction of Mycobacterium tuberculosis Infection via Regulating Their Self-Survival, Differentiation and Metabolism. Front. Immunol..

[29] Shete A., Bichare S., Pujari V., Virkar R., Thakar M., Ghate M., Patil S., Vyakarnam A., Gangakhedkar R., Bai G. (2020). Elevated Levels of Galectin-9 but Not Osteopontin in HIV and Tuberculosis Infections Indicate Their Roles in Detecting MTB Infection in HIV Infected Individuals. Front. Microbiol..

[30] Dunsmore G., Rosero E.P., Shahbaz S., Santer D.M., Jovel J., Lacy P., Houston S., Elahi S. (2021). Neutrophils promote T-cell activation through the regulated release of CD44-bound Galectin-9 from the cell surface during HIV infection. PLoS Biol..

[31] Horio Y., Ichiyasu H., Kojima K., Saita N., Migiyama Y., Iriki T., Fujii K., Niki T., Hirashima M., Kohrogi H. (2017). Protective effect of Galectin-9 in murine model of lung emphysema: Involvement of neutrophil migration and MMP-9 production. PLoS ONE.

[32] Khambati N., Olbrich L., Ellner J., Salgame P., Song R., Bijker E.M. (2021). Host-Based Biomarkers in Saliva for the Diagnosis of Pulmonary Tuberculosis in Children: A Mini-Review. Front. Pediatr..

[33] Shete A., Ghate M., Iwasaki-Hozumi H., Patil S., Shidhaye P., Bai G., Matsuba T., Pharande P., Mahajan B., Randive A. (2024). Dynamics of Matricellular Protein Levels in Blood Predict Recovery in Patients with Human Immunodeficiency Virus-Tuberculosis Coinfection. Viruses.

[34] Shete A., Ghate M., Iwasaki-Hozumi H., Patil S., Shidhaye P., Matsuba T., Bai G., Pharande P., Hattori T. (2024). Association of SARS-CoV-2 Seropositivity with Persistent Immune Activation in HIV/Tuberculosis Co-Infected Patients. Reports.

